# Targeting UAP1 with rabeprazole: a novel repurposed therapeutic strategy for multiple myeloma

**DOI:** 10.3389/fphar.2026.1884338

**Published:** 2026-09-23

**Authors:** Longhao Cheng, Hu Cheng, Bing Hu, Aijun Liu, Xiaoxue Wang, Ying Zhu, Lili Gong, Lihong Liu

**Affiliations:** 1 Institute of Clinical Medical Sciences, China-Japan Friendship Hospital, Capital Medical University, Beijing, China; 2 Department of Pharmacy, The First Affiliated Hospital of Anhui Medical University, Hefei, Anhui, China; 3 Beijing Chao-Yang Hospital, Capital Medical University, Beijing, China; 4 Department of Pharmacy, China-Japan Friendship Hospital, Beijing, China

**Keywords:** cancer treatment, multiple myeloma, rabeprazole, target, UAP1

## Abstract

**Background:**

Despite significant advancements in proteasome inhibitors and immunomodulatory drugs, multiple myeloma (MM) remains largely incurable due to persistent drug resistance and frequent relapse, highlighting an urgent need for novel therapeutic targets.

**Methods:**

We analyzed Multiple Myeloma Research Foundation and GEO datasets to identify genes associated with disease progression, prioritizing UDP-N-acetylglucosamine pyrophosphorylase 1 (UAP1) for functional validation. The impact of UAP1 on MM cell growth was evaluated through both *in vitro* and *in vivo* models. Integrated metabolomics and transcriptomics were employed to elucidate the underlying regulatory mechanisms. Furthermore, virtual screening of an FDA-approved drug library was performed to identify potential UAP1 expression suppressor, followed by experimental validation of the lead compound’s efficacy *via* molecular docking, combination therapy analysis, and gene expression intervention.

**Results:**

UAP1 was found to be overexpressed in MM patients and positively correlated with disease severity. UAP1 knockdown significantly suppressed MM cell proliferation, and promoted apoptosis. Mechanistically, integrated multi-omics analysis revealed that this growth inhibition was driven by the dysregulation of protein biosynthesis, degradation, and amino acid metabolism. Virtual screening identified Rabeprazole (RAB) as a potent expression suppressor candidate. RAB effectively inhibited MM cell growth by downregulating UAP1 expression and exhibited synergistic anti-myeloma effects when combined with the proteasome inhibitor bortezomib. Knockdown and overexpression experiments confirmed that UAP1 is a functional target of RAB.

**Conclusion:**

Our findings establish UAP1 as a critical driver of MM progression and a promising therapeutic target. Genetic or pharmacological inhibition of UAP1 expression *via* RAB disrupts essential metabolic pathways, thereby stunting tumor growth. This study provides preclinical evidence for repurposing RAB as a novel UAP1-targeted strategy, offering a potential avenue to sensitize the therapeutic efficacy of bortezomib and improve clinical outcomes for MM patients.

## 1 Introduction

Multiple myeloma (MM) is characterized by the malignant proliferation of plasma cells within the bone marrow, representing a significant hematological malignancy. Patients typically present with osteolytic lesions, hypercalcemia, anemia, bone pain, and renal impairment due to aberrant plasma cell activation (van de Donk et al., 2021). Epidemiological studies indicate that MM accounts for approximately 1% of all cancer cases and 10% of hematological malignancies, positioning it as the second most prevalent hematological malignancy after non-Hodgkin lymphoma (Rajkumar, 2022). MM primarily affects older adults, with the majority of cases occurring in individuals aged 60–70 years. Notably, the mortality rate exceeds 80% in patients aged 65 and above (Kalariya et al., 2024). As the aging population in China continues to grow, the incidence and mortality associated with MM are expected to rise correspondingly (Dou et al., 2025). Consequently, MM imposes a substantial healthcare burden on patients and the healthcare system.

Despite therapeutic advances-including novel proteasome inhibitors (e.g., carfilzomib, ixazomib), immunomodulatory drugs (e.g., lenalidomide, pomalidomide), and monoclonal antibodies-MM remains incurable. While these agents have extended patient survival, relapse and drug resistance persist, making disease progression control a critical treatment priority (Cowan et al., 2022). Furthermore, the drug development efforts for MM encounter numerous hurdles, including the high costs with immunotherapies, optimization of administration routes, potential for combination with standard therapies, and issues related to patient tolerance. Therefore, the pursuit of MM-specific therapeutic targets is of paramount importance. Investigating the superficial and underlying relationships between MM and its targets could yield significant insights that are crucial for drug development.

Dysregulation of cellular energy and altered metabolism are recognized hallmarks of cancers (Pavlova and Thompson, 2016). The shift from glucose metabolism to anaerobic glycolysis, glutamine pathways and changes in fatty acid metabolism have been observed in almost all types of tumors. Interestingly, the hexosamine biosynthetic pathway (HBP) also plays a pivotal role in tumor metabolism by regulating the synthesis of key metabolites, particularly UDP-N-acetylglucosamine (UDP-GlcNAc) (Lam et al., 2021). This pathway, which branches off from the glycolytic pathway, is critical for the production of glcnacylation substrates that influence various cellular functions, including protein modification and signaling. In cancer cells, the HBP is often upregulated, leading to increased levels of UDP-GlcNAc, which in turn promotes the O-GlcNAcylation of proteins. This post-translational modification has been implicated in the modulation of oncogenic signaling pathways, cellular proliferation, and resistance to apoptosis (Wu et al., 2021). Furthermore, the HBP intersects with other metabolic pathways, establishing a complex network that supports the altered metabolic demands of tumor cells (Bi et al., 2020; Loison et al., 2024). The HBP is highly activated in MM. Recent evidence identifies the HBP as a core pathway driving drug resistance, and its activity is indispensable for mitochondrial adaptation. Targeting the HBP axis disrupts the interconnected mitochondrial-glycometabolic network and restores the sensitivity of bortezomib (BTZ)-resistant MM cells (Tibullo et al., 2020). Consequently, targeting the HBP may offer novel therapeutic strategies for disrupting the metabolic adaptations of MM, highlighting its significance in the tumor progression.

At the core of the HBP, UDP-N-acetylglucosamine pyrophosphorylase 1 (UAP1) functions as a key enzyme, catalyzing the conversion of fructose-6-phosphate into UDP-GlcNAc8. This enzymatic activity positions UAP1 as a crucial regulator of the HBP. Dysregulation of UAP1 expression is often observed in cancer, leading to heightened UDP-GlcNAc levels and subsequent alterations in protein O-GlcNAcylation. For instance, UAP1 is highly expressed in prostate cancer, resulting in a 10-fold increase in UDP-GlcNAc levels. This elevation in UAP1 is associated with resistance to N-glycosylation inhibitors and protects prostate cancer cells from endoplasmic reticulum stress (Itkonen et al., 2015). Additionally, UAP1 has been shown to be overexpressed in lung adenocarcinoma, correlating with poor patient prognosis (Wang et al., 2020). Consequently, UAP1 is considered a potential biomarker with significant clinical implications for disease diagnosis and treatment. Understanding the interplay between UAP1 and the HBP not only elucidates the metabolic adaptations of cancer cells but also underscores the potential of targeting UAP1 in therapeutic strategies. However, the role of UAP1 in MM remains unclear, and it is still ambiguous whether the UAP1-mediated HBP plays a role in MM. Therefore, further investigation is necessary to determine whether UAP1 could serve as a potential therapeutic target in MM.

Molecular targeted therapy represents a transformative approach in oncology, selectively inhibiting tumor development and metastasis or inducing apoptosis while sparing adjacent normal tissues, thereby offering superior efficacy and reduced adverse effects compared to conventional treatments (Bedard et al., 2020; Lee et al., 2018). These advantages underscore the critical importance of identifying reliable prognostic markers, novel therapeutic targets, and targeted agents for MM. Computational approaches, especially molecular docking technology, provide powerful solutions for target-based drug discovery. This methodology predicts ligand-receptor binding affinity and interaction modes through biomolecular simulations, enabling efficient virtual screening of compound libraries against defined targets. Such *in silico* strategies substantially decrease the time, financial investment, and experimental resources required for lead compound identification. Consequently, molecular docking accelerates the development of novel target candidates and optimizes drug discovery for therapeutics (Dong et al., 2018; Suay-García et al., 2022).

This study utilized a bioinformatics approach to identify the key gene UAP1, which is associated with the development and progression of MM. The role of UAP1 was subsequently validated at the animal level, and a series of drugs were screened based on this newly discovered target UAP1.

## 2 Materials and methods

### 2.1 Data sources

The transcriptome data of initially treated patients with MM were obtained from the MMRF database, a large-scale prospective study of newly diagnosed MM that includes information from 859 patients from more than 90 different sites worldwide. Tumor cell transcriptome data were obtained from the CCLE, which contains 1,072 cell lines from diverse lineages and ethnicities. Transcriptome data from healthy people were obtained from the Genotype-Tissue Expression (GTEx) database. The downloaded datasets GSE6477 (GPL96), GSE13591 (GPL96), and GSE57317 (GPL570) contain 375 MM samples.

### 2.2 Clinical samples

Bone marrow (BM) aspirates were collected from 18 participants, including 10 non-malignant control individuals and eight patients with high-risk MM. BM aspiration was performed for all subjects under routine procedures. BM samples were extracted in the early morning on an empty stomach and stored at −80 °C until pre-treatment. All clinical samples were obtained from Beijing Chaoyang Hospital. Patients with high-risk MM were diagnosed in accordance with the diagnostic criteria in the guidelines of the International Myeloma Working Group. This study was approved by the Research Ethics Committee of Beijing Chaoyang Hospital (No. 2021-3-22-3). This research was carried out in accordance with the Declaration of Helsinki’s guidelines and all applicable laws regarding the use of human study subjects. After red blood cells lysis of the BM samples, the CD138^+^ MM cells were isolated and stored in Trizol reagent (Life Invitrogen, United States). Metabolomic analysis of clinical MM samples was carried out as described by Cheng et al. (2024). Briefly, a total of 176 plasma samples, including 33 healthy controls and 143 newly diagnosed MM patients were collected. Another 114 BM samples (47 non-malignant controls and 67 newly diagnosed MM patients) were included. HBP-related metabolites were extracted from the metabolomic data, and semi-quantified by peak areas to assess HBP changes in MM.

### 2.3 Cell culture

Cell lines KMY1022, NCI-H929, RPMI-8226, LAMA-84 and ARD were obtained from the Cell Resource Center (IBMS, CAMS/PUMC, China), and cultured in RPMI 1640 medium supplemented with 10% FBS, penicillin (100 U/mL) and streptomycin (100 μg/mL) in an atmosphere with 5% CO_2_ at 37 °C. These cells are mycoplasma-free.

### 2.4 Cell viability and cell proliferation

Cell viability was assessed using the CCK-8 assay (Livning, China). Approximately 4 × 10^4^ cells were seeded into each well of a 96-well plate, with a total volume of 100 µL per well, and incubated for 48 h. Subsequently, 10 µL of CCK-8 reagent was added, and the cells were incubated in the dark at 37 °C for 2 h. The absorbance was then measured at a wavelength of 450 nm using a microplate reader. Combination index (CI) analysis was performed and CI values were calculated using CompuSyn software. Cell proliferation was evaluated using the EdU proliferation assay kit (Beyotime, China), following the manufacturer’s instructions for all steps. Approximately 4 × 10^5^ cells were seeded into each well of a 6-well plate, with a total volume of 2 mL per well, and incubated for 48 h. After labeling the MM cells with fluorescence, images were captured using an Olympus fluorescence microscope.

### 2.5 Flow cytometry analysis of cell cycle and apoptosis

Cell cycle analysis was conducted using a propidium iodide (PI) assay kit (Keygen, China), following the manufacturer’s instructions for all steps. Briefly, MM cells were fixed and subsequently incubated with RNase/PI staining working solution. The DNA content was then analyzed using flow cytometry at an excitation wavelength of 488 nm to assess DNA replication. Apoptosis was evaluated using a Annexin V-PE/7-AAD assay kit (BD Bioscience, United States), adhering to the manufacturer’s guidelines. Briefly, MM cells were incubated with Annexin V-PE and 7-AAD dyes, and the proportion of apoptotic cells was analyzed and quantified using flow cytometry.

### 2.6 Cell transfection

MM cells were seeded in 6-well plates 24 hours prior to transfection. After diluting the siRNAs (30 pmol) and Lipofectamine™ RNAiMAX Transfection Reagent (Thermo Fisher Scientific, United States), the mixture was gently mixed and added to the cells. Following a 48-h transfection period, cell RNA was collected, while cell protein was harvested after 72 h of transfection. All siRNAs were obtained from Beijing Syngentech Co., Ltd. and included four distinct sequences targeting the human UAP1 gene [siNC: sense 5′-UUC​UCC​GAA​CGU​GUC​ACG​U-3′, antisense 5′-ACG​UGA​CAC​GUU​CGG​AGA​A-3’; siUAP1-① (the most effective sequence): sense 5′-GGU​AUU​AGG​CAG​UGC​UAC​A-3′, antisense 5′-UGU​AGC​ACU​GCC​UAA​UAC​C-3’; siUAP1-②: sense 5′-CAA​UGA​UGU​ACC​AAU​CCA​A-3′, antisense 5′-UUG​GAU​UGG​UAC​AUC​AUU​G-3’; siUAP1-③: sense 5′-CUA​UUG​UGU​UGA​CAA​CAU​A-3′, antisense 5′-UAU​GUU​GUC​AAC​ACA​AUA​G-3’]. A negative control siRNA was also included in the experiment. For plasmid transfection, the overexpression plasmid was constructed by cloning the full-length coding sequence of human UAP1 gene (NM_003115.5) into the pCMV3 vector, and the corresponding empty vector was used as the negative control. Plasmids and Lipofectamine 3,000 Reagent were diluted separately, gently mixed to form a transfection complex, and then added to the cells. The transfection duration was 72 h. The overexpression plasmids and corresponding empty vector control were purchased from Beijing Syngentech Co., Ltd.

### 2.7 Real-time qPCR analysis

Total RNA was extracted from MM cell lines using Trizol reagent (Life Invitrogen, United States), and reverse transcribed into cDNA using a cDNA Synthesis Kit (Tiangen, China), according to the manufacturer’s instructions. Real-time qPCR was performed using SuperReal PreMix Plus SYBR Green kit (Tiangen, China). The specific primers used for qPCR were as follows: *UAP1* forward primer: 5′-GGG​ATC​AAG​ATC​AGC​TCC​AG-3′, reverse primer: 5′-GAT​ATG​CAA​CGC​CGA​GTC​TT-3’; *ACTB* forward primer: 5′-CTC​TTC​CAG​CCT​TCC​TTC​CT-3′, reverse primer: 5′-AGC​ACT​GTG​TTG​GCG​TAC​AG-3’. The expression levels of the target genes were calculated and quantified using comparative CT (2^−ΔΔCT^) method.

### 2.8 Western blot

The protein extraction from MM cells and tumor tissue protein of subcutaneous transplanted tumor model were extracted according to the manufacturer’s instructions. The protein concentration was quantified with the BCA kit. Western blot analysis was performed using SDS-PAGE gels. Electrophoresis was performed first at 80 V for 30 min, followed by 120 V for 90 min, and membrane rotation at 400 mA for 120 min. The membranes were then blocked with 5% skim milk and incubated with primary and secondary antibodies. The band strength was analyzed by a gel documentation system. Row values were quantified by ImageJ to calculate the relative protein abundance. Primary antibodies used were as follows: anti-β-Actin (Cell Signaling Technology, 4967, 1:5,000), anti-UAP1 (Proteintech, 16318-1-AP, 1:1,000), anti-O-GlcNAc (Cell Signaling Technology, 82332S, 1:1,000), anti-ATP4A (Proteintech, 55124-1-AP, 1:1,000), anti-ATP4B (Abways, CY8747, 1:1,000), anti-c-MYC (Abways, CY5150, 1:1,000), anti-IRF4 (Abways, CY9827, 1:1,000).

### 2.9 Subcutaneous xenografts

The shRNA lentiviral vector was used to knockdown UAP1 in NCI-H929 cells (UAP1-KD). The lentivirus was transfected into the cells, and puromycin at a concentration of 10 μg/mL. The stable mixed clone cells were diluted and cultured at 24 h. The cell clones grown from a single cell were selected, and then expanded and cultured to obtain a cell line with single character and stable expression. The UAP1 knockdown lentiviruses were purchased from Beijing Syngentech Co., Ltd. The NOD.Cg-PrkdcscidIL2rgtm1Sug/JicCrl (NOG) male mice used in the animal experiments were purchased from Beijing Vital River Laboratory Animal Technology Co., Ltd. Each six-week-old male NOG mouse was inoculated with 6.5 ×10^6^ cells. Mice were randomly assigned to experimental groups (n = 5 per group). The sample size was determined based on a power analysis (80% power, α = 0.05), assuming a minimum difference in tumor volume of 30% and a standard deviation of 15% based on preliminary studies. The animal experiments were terminated on day 21 after inoculation. All procedures involving animal studies were ethically approved by the China-Japan Friendship Hospital Institutional Animal Ethics Committee.

### 2.10 Metabolites extraction and untargeted metabolomics analysis

About 50 mg of each tumor tissue from animal experiments was placed in 1.5 mL tubes, and homogenized using 10 times the volume of 50% methanol-aqueous solution. The homogenates were then centrifuged at 12,000 rpm for 10 min, then 50 μL of the supernatant was transferred to a new tube. The pre-treatment solution was added, and the supernatant was mixed for 2 min, centrifuged at 12,000 rpm for 10 min, and the supernatant was collected as the sample for further analysis.

An ultra-performance liquid chromatography and a high-resolution mass spectrometer QE-Orbitrap (UPLC-HRMS) were employed for detection (Thermo Fisher Scientific, United States). Detailed methods for UPLC-HRMS-based untargeted metabolomics were performed as described in Cheng et al. (2024).

Raw MS data were processed using Compound Discoverer 3.1 software (Thermo Fisher Scientific, United States) for peak picking, retention time alignment, peak deconvolution, and noise reduction. Metabolite identification was achieved by matching the accurate mass, retention time, and fragmentation patterns with public databases including Human Metabolome Database, METLIN, and Lipid Maps, with a mass deviation ≤10 ppm. Raw peak intensities were normalized to eliminate systematic variations between samples. Multivariate statistical analysis (principal component analysis, PCA, partial least squares discrimination analysis, OPLS-DA, and heatmap) were carried out to identify remarkable metabolic features using MetaboAnalyst 6.0 (https://www.metaboanalyst.ca/). Differential metabolites were screened based on the criteria of Variable Importance in Projection (VIP) > 1 from the OPLS-DA model and P < 0.05 from unpaired two-sided t-tests. The metabolic pathway analysis was obtained using the module “MS Peaks to Pathways” of MetaboAnalyst 6.0. Integrative metabolomics and transcriptomics analysis was performed using the “Pathway Analysis” and “Network Analysis” modules in MetaboAnalyst 6.0.

### 2.11 LC-MS quantification targeted UDP-hex

MM cells (1 × 10^7^ cells/mL) cells were collected and washed three times with PBS. The cells were then resuspended in 50% methanol and vortexed, followed by ultrasonication to precipitate the proteins. The supernatant was collected and diluted with a regent containing internal standards (methanol:acetonitrile = 1:1, with propranolol at 100 ng/mL and tolbutamide at 200 ng/mL) before quantitative analysis. The injection sequence for all samples was randomized to minimize bias. Given the difficulty in differentiating UDP-GlcNAc and UDP-GalNAc in the total ion chromatogram (TIC), the results were analyzed based on the peak area of UDP-hex, representing the combined levels of UDP-GlcNAc and UDP-GalNAc.

For LC conditions, a ZIC-pHILIC column (150 mm × 2.1 mm) was utilized, with a column temperature maintained at 40 °C. The mobile phase consisted of A: a 20 mM ammonium carbonate solution with 0.1% ammonium hydroxide, and B: acetonitrile, with a flow rate set at 0.2 mL/min. Additional LC and MS conditions were implemented as previously described by Mason et al. (2020).

### 2.12 Quantification of intracellular UDP-GlcNAc content

NCI-H929 cells were harvested and counted. After thorough cell lysis, subsequent experimental procedures were performed strictly following the manufacturer’s instructions of the Human UDP-N-acetyl glucosamine ELISA Kit (JILID, China, J22163). Briefly, cell lysate supernatants were collected. Appropriate sample diluent and cell lysate were added to microplate wells for a 5-fold sample dilution. HRP-conjugated reagent was supplemented to all wells except blank wells, followed by 60 min incubation at 37 °C. After washing, chromogen solutions A and B were added for color reaction. The reaction was terminated with stop solution, and absorbance at 450 nm was measured. The intracellular UDP-GlcNAc concentrations were calculated according to the standard curve, and the concentration was normalized to cell density.

### 2.13 RNA-seq analysis

Total RNA was extracted from subcutaneous tumor tissue of mice using the Trizol Reagent (Life Invitrogen, United States). The extracted RNA was assessed for concentration, quality and integrity using a NanoDrop spectrophotometer (Thermo Fisher Scientific, United States). A total of 3 μg of RNA were used for the RNA sample preparations.

Sequencing libraries were generated according to the following steps. Firstly, mRNA was purified from total RNA using poly-T oligo-attached magnetic beads. Fragmentation was carried out using divalent cations under elevated temperature in an Illumina proprietary fragmentation buffer. First strand cDNA was synthesized using random oligonucleotides and Super Script II. Second strand cDNA synthesis was subsequently performed using DNA Polymerase I and RNase H. Remaining overhangs were converted into blunt ends *via* exonuclease/polymerase activities and the enzymes were removed. After adenylation of the 3′ ends of the DNA fragments, Illumina PE adapter oligonucleotides were ligated to prepare for hybridization. To select cDNA fragments of the preferred 400–500 bp in length, the library fragments were purified using the AMPure XP system (Beckman Coulter, United States). DNA fragments with ligated adaptor molecules on both ends were selectively enriched using Illumina PCR Primer Cocktail in a 15 cycle PCR reaction. Products were purified (AMPure XP system) and quantified using the Agilent high sensitivity DNA assay on a Bioanalyzer 2100 system (Agilent, United States). The sequencing library was then sequenced on NovaSEquation 6,000 platform (Illumina, United States) by Shanghai Personal Biotechnology Co. Ltd. Differential gene expression analysis was performed using the DESeq2 R package. Genes with |fold change| > 5 and adjusted P < 0.05 were identified as differentially expressed genes. GO enrichment analysis included biological process, cellular component, and molecular function categories, with enrichment significance determined by P < 0.05. KEGG pathway enrichment analysis was performed using the KEGG database, and significantly enriched pathways were defined as P < 0.05. GSEA was conducted to explore the enrichment of Hallmark gene sets, with 1,000 permutations. Heatmaps and enrichment plots were generated using the ggplot2 and enrichplot R packages, respectively.

### 2.14 Molecular docking drug screening based on UAP1

The UAP1 protein structure (ID: 1VM8) was retrieved from the Protein Data Bank. Compound libraries were sourced from DrugBank. Protein preparation involved: (1) removing crystallographic water molecules, (2) eliminating alternate conformations, and (3) repairing missing residues using Discovery Studio’s “Prepare Protein” module. Ligands were prepared *via* the “Prepare Ligands” module to generate valid 3D conformations. Molecular docking was performed with the “LibDock” module, defining UAP1’s ligand-binding pocket as the active site. The LibDock Score evaluated protein-ligand binding affinity. Top-ranking compounds were selected based on docking scores. Binding modes and interaction patterns (hydrogen bonds, hydrophobic contacts) were visualized and analyzed using the “Analyze Results” module. Selected candidates underwent *in vitro* validation (MM cell proliferation assays) and *in vivo* testing (xenograft models) to confirm UAP1 expression suppressor activity.

### 2.15 Surface plasmon resonance (SPR) analysis

SPR were performed using a Biacore 1K instrument (Cytiva, Sweden). UAP1 was covalently immobilized as the ligand onto the surface of a CM5 sensor chip *via* standard amine coupling. Briefly, the chip surface was activated with a mixture of EDC and NHS, followed by the injection of UAP1; unreacted active sites were subsequently blocked with ethanolamine. Rabeprazole (RAB) was prepared as the analyte and serially diluted in HBS-EP + running buffer to seven concentration gradients: 0, 2.5, 5, 10, 20, 40, and 80 μM.After the chip reached a stable baseline in the running buffer, samples were injected in increasing order of concentration. For each cycle, an injection volume of 200 μL was used at a flow rate of 20–30 μL/min. Association, dissociation, and regeneration steps were completed to capture the response signals. Given the characteristically slow binding kinetics of the small molecule-protein interaction, data analysis was conducted using a 1:1 binding model and steady-state affinity fitting *via* the Biacore Evaluation software to determine the equilibrium dissociation constant (KD value). To ensure the reliability of the experimental results, non-specific binding was verified to be negligible (response near zero at 0 μM), and the baseline drift was maintained at < 0.5 RU/minute.

### 2.16 Co-immunoprecipitation (Co-IP)

NCI-H929 cells were treated with RAB (10 μM) for 48 h, then lysed in IP buffer containing protease/phosphatase inhibitors. Cell lysates were incubated with anti-c-MYC or anti-IRF4 antibody overnight at 4 °C, followed by precipitation with Protein A/G magnetic beads for 2 h. Beads were washed with IP buffer, and immunoprecipitated proteins were analyzed by Western blot using anti-O-GlcNAc antibody to assess O-GlcNAcylation levels of c-MYC and IRF4.

### 2.17 Statistical analysis

The experiments were biologically repeated three times. All data were presented as means ± SEM. Unpaired two-sided t-tests were used to compare two experimental groups, while one-way ANOVA tests were utilized for comparing three or more experimental groups. P < 0.05 was considered statistically significant. The analysis was performed using GraphPad Prism 8.0.

## 3 Results

### 3.1 Expression profile of UAP1 in MM databases

In this study, we conducted a comparative analysis of transcriptomic data from MM patients sourced from the Multiple Myeloma Research Foundation (MMRF) and Encyclopedia of Cancer Cell Lines (CCLE) databases against control populations, visualizing the results with volcano plots. Utilizing the Robust Rank Aggregation algorithm, we identified differentially expressed genes. Through cross-analysis, we integrated the differentially expressed genes from both datasets, ultimately identifying a consensus of 109 shared differentially expressed genes. Subsequently, we analyzed and validated these 109 differentially expressed genes using data from the MMRF and GEO databases, narrowing our focus to 19 candidate genes, which included UAP1, ATP8B2, AQP3, CADPS2, JCHAIN, BMP8B, and BMP6 (Figure 1A). To elucidate the relationship between these differentially expressed genes and MM progression as well as poor prognosis, we employed R packages to analyze gene expression levels in the GEO database and constructed survival curves. Our findings indicated that high expression levels of UAP1 are associated with significantly shorter survival times in MM patients, whereas low UAP1 expression correlates with prolonged survival (Figure 1B). Furthermore, we validated UAP1 expression across various datasets within the GEO, revealing that UAP1 not only exhibits elevated levels in MM patients compared to controls (Figure 1C) but also shows a progressive increase correlating with the advancement of the disease stage (Figures 1D–F). Except for UAP1, the expression levels of other genes do not exhibit a significant correlation with the progression of MM (Supplementary Figures S1–S6). They were not established as reliable prognostic biomarkers for patient survival outcomes. Therefore, these genes were excluded following a comprehensive screening and assessment process. Above all, these results underscore the aberrant expression of UAP1 in MM and highlight its potential significance as a biomarker for disease progression and prognosis, warranting further investigation into its mechanistic role in MM.

**FIGURE 1:**
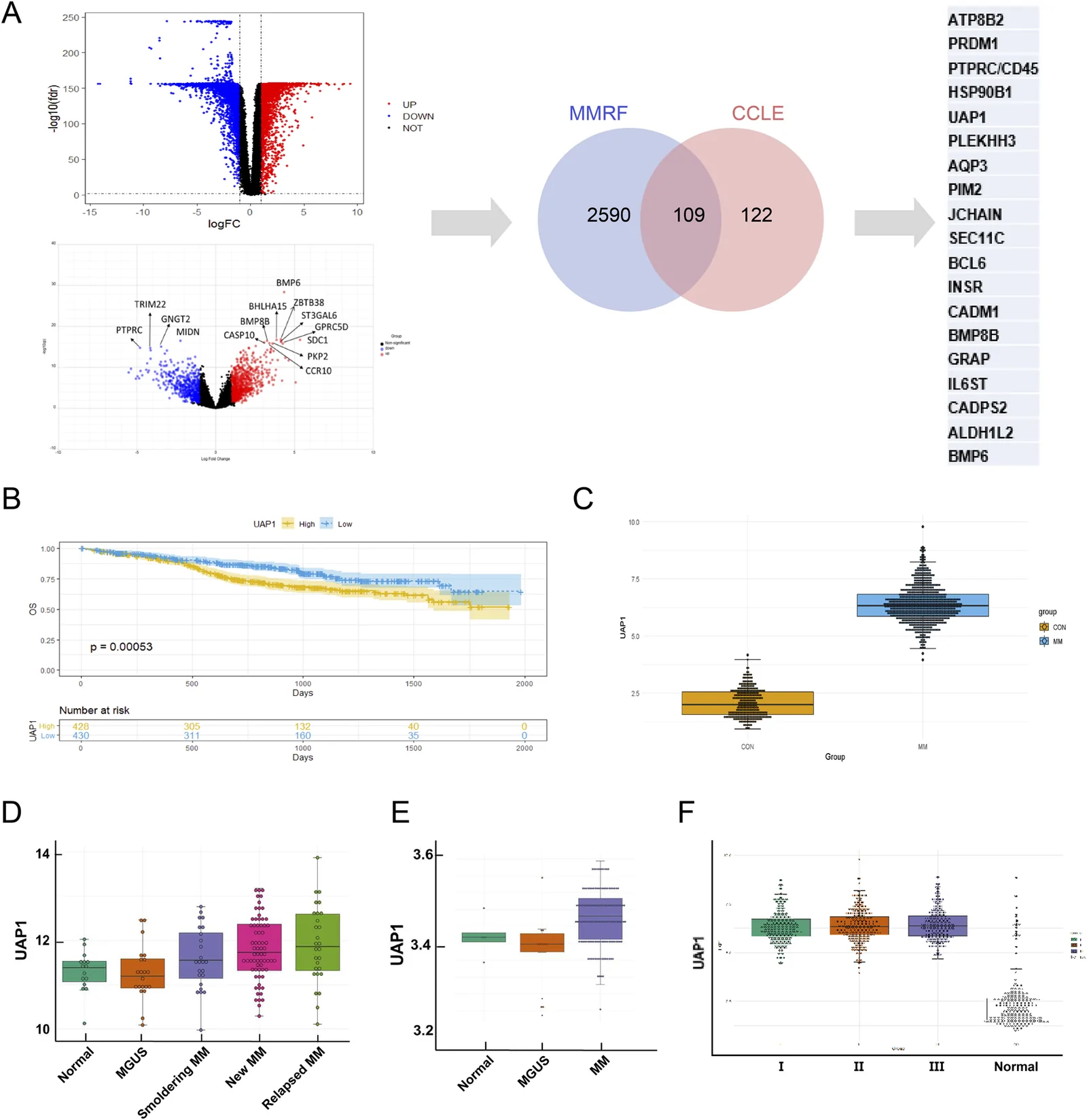
Screening of differential targets of MM from public databases. **(A)** Schematic diagram of MMRF and CCLE database analysis and potential target screening. **(B)** MMRF database showed the relationship between UAP1 expression and patient survival, and the survival of the UAP1 high expression group was significantly lower than that of the low expression group, P < 0.001. **(C)** CCLE database demonstrated the expression of UAP1 in the control population and MM patients. **(D–F)** GEO database analyzed the expression of UAP1 in different stages and phases of MM.

### 3.2 UAP1 is upregulated in MM and regulates the O-GlcNAc levels

To further elucidate the expression of UAP1 in MM, we first examined the collected BM samples from MM patients. We found that the expression of the *UAP1* gene was significantly upregulated in the MM group, a finding consistent with the analyses of public databases (Figure 2A). We subsequently conducted a comparative analysis across various MM cell lines. Our findings revealed that UAP1 expression levels varied significantly among the different cell lines when compared to normal B cells. Only the LAMA-84 and MM.1S cell lines exhibited low expression levels, while other MM cell lines showed markedly elevated UAP1 expression (Figures 2B,C). UAP1 functions as the terminal enzyme in the HBP, which is crucial for the intracellular synthesis of UDP-GlcNAc. An increase in UDP-GlcNAc levels promotes the synthesis of O-GlcNAc, a post-translational modification of proteins. Consequently, elevated UAP1 expression may directly influence O-GlcNAc levels within the cells. To assess this potential effect, we measured the expression of O-GlcNAc proteins, which were found to be highly expressed in MM cells (Figure 2C). To further validate the global activation of the HBP, we examined core substrates using clinical metabolomic data. L-Glutamine, the key nitrogen donor of the HBP, was depleted in BM cells from MM patients. Levels of D-Glucosamine and GlcNAc, which feed into the HBP salvage pathway, were also reduced (Figure 2D). Conversely, peripheral blood levels of L-Glutamine and GlcNAc were elevated, suggesting that UAP1-driven HBP activation increases substrate demand in the BM niche, which is replenished by supply from the peripheral circulation (Figure 2E). As UDP-GlcNAc and UDP-GalNAc are C4 epimers, GlcNAc and GalNAc are interconvertible, resulting in inverse changes in their abundances. Nevertheless, their nearly identical structures produce overlapping peak profiles, making them difficult to resolve by mass spectrometry. Therefore, we utilized UDP-hexosamines (UDP-hex) as a collective term for both metabolites (Figure 2F). Quantitative analysis revealed a decrease in the levels of UDP-hex within MM cells, suggesting that UDP-GlcNAc and UDP-GalNAc are increasingly utilized for O-GlcNAc synthesis (Figure 2G). We hypothesize that this results in elevated levels of protein O-GlcNAc modification in MM cells. Additionally, when UAP1 expression was inhibited, we observed a suppression of O-GlcNAc expression alongside an increase in intracellular UDP-hex (Figures 2H,I). These results collectively indicate that UAP1 is highly expressed in MM cells. Notably, high UAP1 expression was associated with lower UDP-hex levels but positively correlated with O-GlcNAc modification.

**FIGURE 2:**
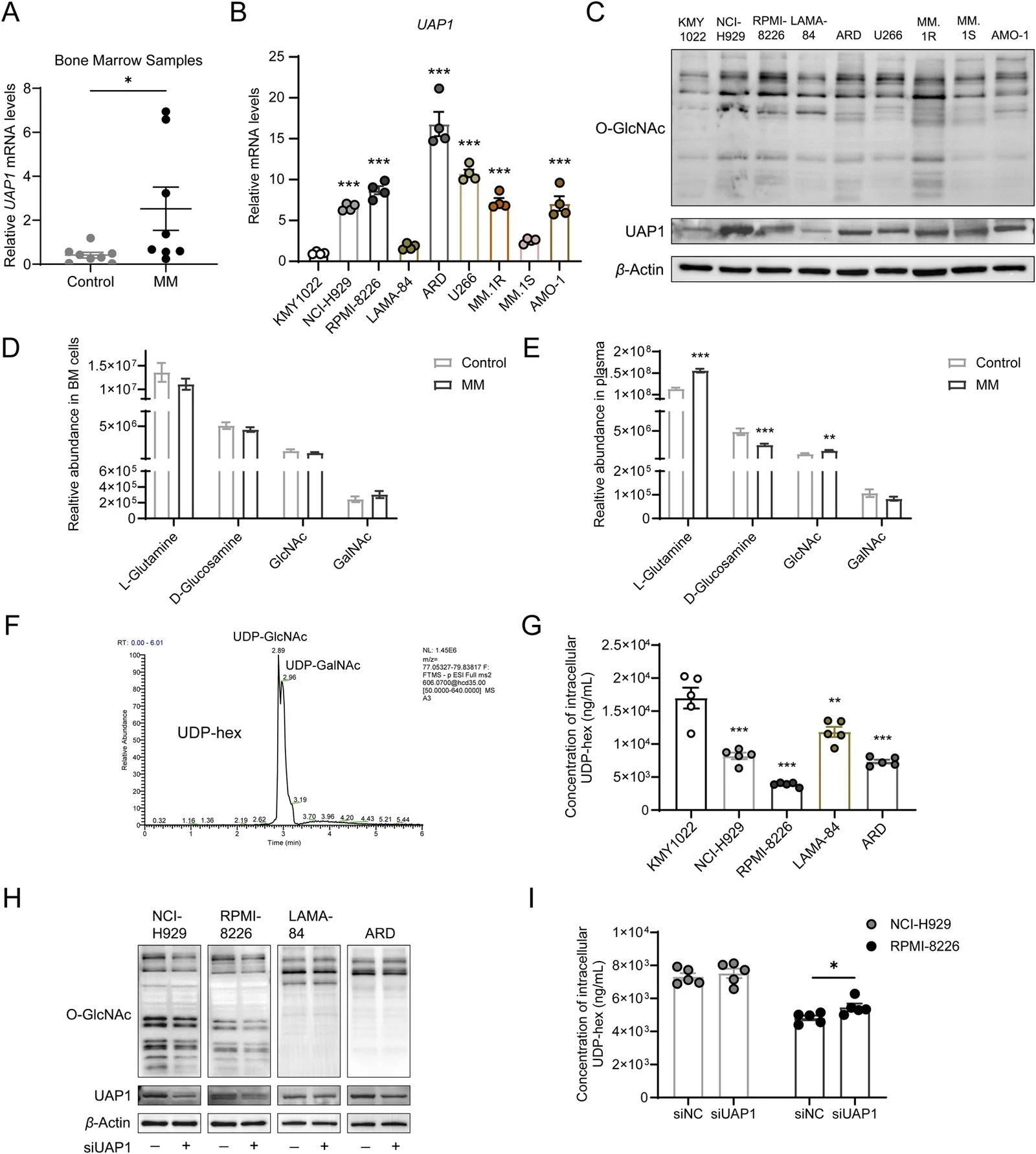
UAP1 expression and O-GlcNAcylation level in MM patients and cell lines. **(A)** mRNA expression levels of *UAP1* in BM samples compared MM patients and control population. **(B)** mRNA expression levels of *UAP1* in various MM cell lines. **(C)** Protein expression levels of O-GlcNAc and UAP1 in different MM cell lines. **(D)** Alterations in the levels of HBP-related metabolites in the plasma of MM patients from metabolomic analysis. **(E)** Alterations in the levels of HBP-related metabolites in bone marrow CD138^+^ cells of MM patients from metabolomic analysis. **(F)** UDP-GlcNAc and UDP-GalNAc belong to the C4 epimers, which can be differentiated in the TIC, and UDP-hex was used to indicate the overall level of UDP-GlcNAc and UDP-GalNAc. **(G)** Intracellular concentrations of UDP-hex in various MM cell lines. **(H)** Changes in the expression levels of O-GlcNAc and UAP1 proteins following siUAP1 in NCI-H929 and RPMI-8226 cells. **(I)** Intracellular levels of UDP-hex following siUAP1 in NCI-H929 and RPMI-8226 cells. Data are shown as mean ± SEM; n ≥ 3 *P < 0.05, **P < 0.01, ***P < 0.001, as assessed with an unpaired two-sided t-test and a one-way ANOVA test.

### 3.3 UAP1 expression promoted MM cell growth and inhibited cell apoptosis *in vitro*

To elucidate the critical role of UAP1 in MM cells, we performed an expression intervention targeting UAP1. Following siUAP1 treatment, we observed that UAP1 plays a role in the proliferation of MM cells; specifically, UAP1 knockdown resulted in a reduction in cell proliferation rates (Figure 3A). Additionally, silencing UAP1 resulted in a significant cell cycle arrest at the G2/M phase, accompanied by a decrease in the number of cells in the S phase. In contrast, no significant cell cycle effects were observed in ARD cells following siUAP1 treatment (Figure 3B). Further analysis using flow cytometry revealed an increased proportion of apoptotic cells following UAP1 expression interference, encompassing both early and late apoptotic stages; however, this phenomenon was not observed in ARD cells (Figure 3C). Collectively, these findings suggest that the elevated expression of UAP1 in MM cells promotes cell growth, while the downregulation of UAP1 can modestly inhibit proliferation and induce apoptosis in MM cells. This highlights the potential pharmacological antagonism of MM through the modulation of UAP1 expression. It is important to note that the variable expression levels of UAP1 across different MM cell lines influenced the degree of response to siUAP1. Specifically, LAMA-84 cells exhibited low levels of UAP1 expression, while ARD cells demonstrated the highest levels. Consequently, the phenotypic changes observed post-intervention in these 2 cell lines were not pronounced. In contrast, NCI-H929 and RPMI-8226 cells displayed moderate UAP1 expression levels, and siUAP1 treatment significantly affected both proliferation and apoptosis in these cell lines. Notably, the influence of UAP1 on downstream O-GlcNAc levels was particularly observed in NCI-H929 cells. Therefore, we selected NCI-H929 cells for subsequent *in vivo* experiments to further explore the UAP1 function in MM.

**FIGURE 3:**
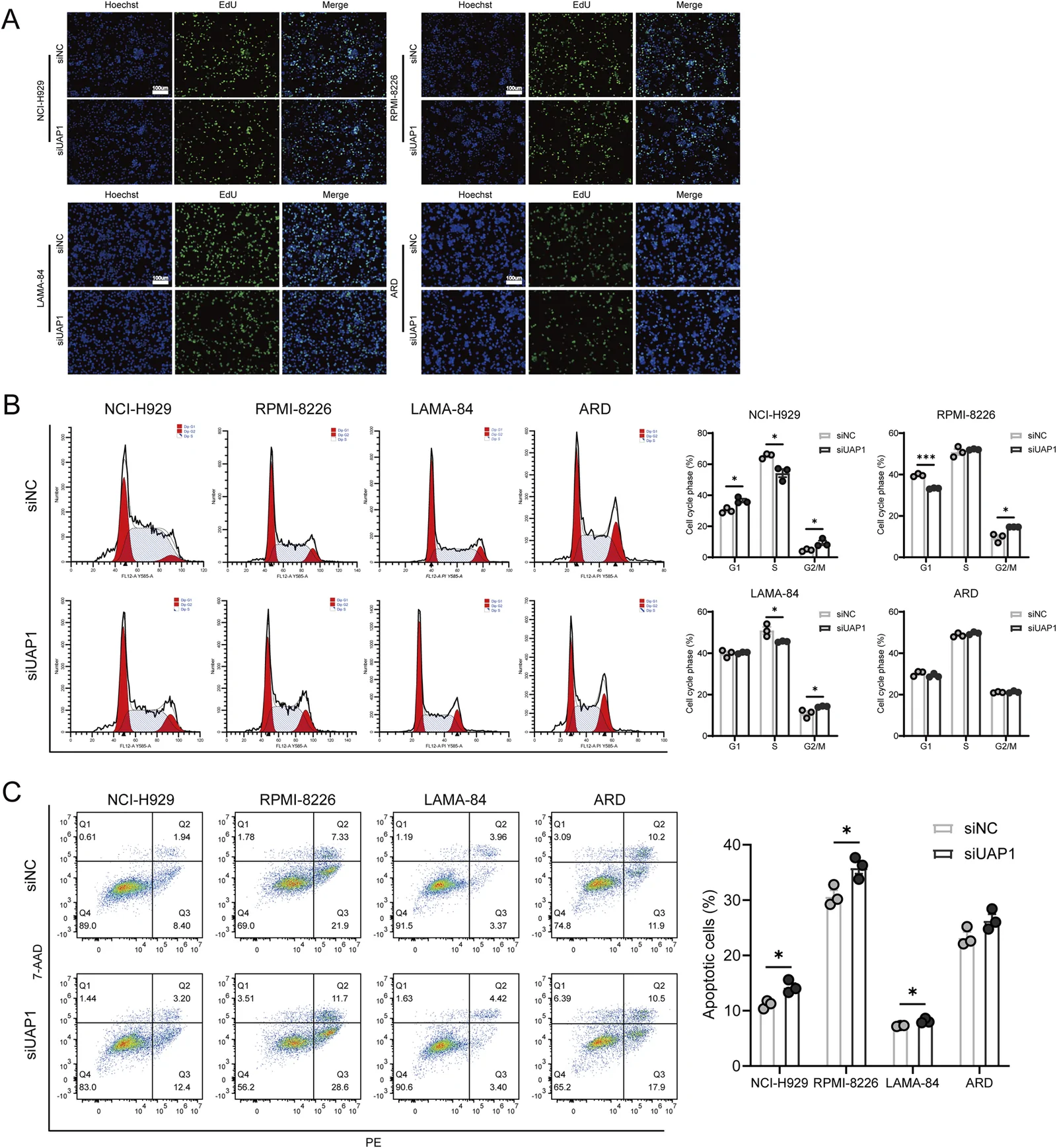
UAP1 expression enhances proliferation of MM cells. **(A)** Cell proliferation of MM cells after siUAP1 was detected by EdU assay. **(B)** Cell cycle of MM cells after siUAP1 was detected by PI assay. **(C)** Apoptosis of MM cells after siUAP1 was detected by PE/7-AAD assay. Data are shown as mean ± SEM; n ≥ 3 *P < 0.05, **P < 0.01, ***P < 0.001, as assessed with an unpaired two-sided t-test.

### 3.4 Metabolomics on the role of UAP1 *in vivo*

To further investigate the role of UAP1 *in vivo*, we utilized lentiviral transfection to establish UAP1 knockdown (KD) in NCI-H929 cells. Subsequently, we implanted UAP1-KD cells into NOG mice to assess the impact of UAP1 knockdown on the tumorigenic capacity of MM cells. Our initial evaluation confirmed an approximate 90% reduction in UAP1 protein expression in the UAP1-KD cells (Figure 4A). After subcutaneously transplanting UAP1-KD cells into NOG mice and allowing a 1-month observation period, we noted a significant suppression in tumor growth rates of NCI-H929 cells, with tumor volumes markedly reduced compared to control groups (Figure 4B). Immunohistochemical analysis validated high expression levels of UAP1 in the control tumors, whereas UAP1 expression was diminished in tumors from UAP1-KD cells (Figure 4C). HE staining revealed notable histopathological alterations in the UAP1-KD group, including tissue vacuolization, increased intercellular spacing, cytoplasmic shrinkage, rupture, and deeply stained nuclei, indicating that reduced UAP1 expression directly affects the *in vivo* status and tumorigenicity of MM cells (Figure 4D).

**FIGURE 4:**
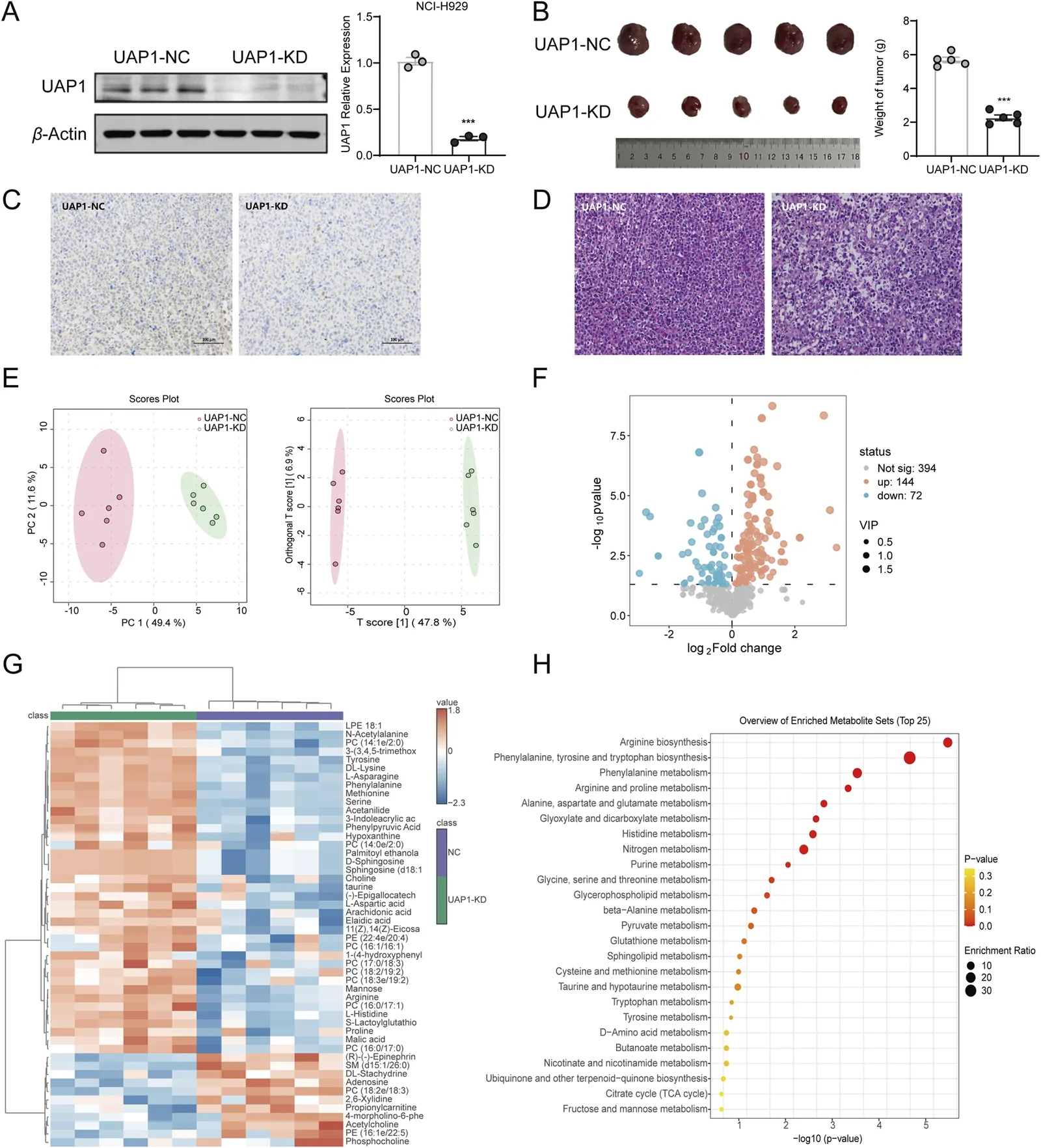
Metabolic mechanism of UAP1-KD suppressing MM tumor growth *in vivo*. **(A)** Western blot analysis of UAP1 protein knockdown in UAP1-KD cells. **(B)** Gross images of tumors in NOG mice after subcutaneous transplantation of normal MM cells and UAP1-KD cells. **(C)** Immunohistochemistry of UAP1 in subcutaneous tumor tissues of NOG mice. **(D)** HE staining of subcutaneous tumor tissues of NOG mice. **(E)** PCA and OPLS-DA of untargeted metabolomic profiling in mouse tumor tissues, comparing UAP1-NC and UAP1-KD groups. **(F)** Volcano plot of the differential metabolites. **(G)** Heatmap of the top 50 metabolites ranked by P-value based on differential metabolomic analysis. **(H)** KEGG enrichment analysis based on the identified differential metabolites. Data are shown as mean ± SEM; n ≥ 3 *P < 0.05, **P < 0.01, ***P < 0.001, as assessed with an unpaired two-sided t-test.

To explore the metabolic mechanisms underlying the inhibition of MM cell growth due to UAP1 knockdown, we conducted metabolomic profiling of tumor tissues from the mice. The metabolomic analysis demonstrated reliability and stability, clear separation between the control and UAP1-KD samples (Figure 4E). This indicated significant differences in metabolites between the groups. Further statistical analysis revealed a total of 144 significantly upregulated metabolites and 72 significantly downregulated metabolites in the volcano plot, which collectively constituted the metabolic alterations induced by UAP1 knockdown (Figure 4F). Cluster analysis of the tumor tissue metabolites identified the top 50 metabolites affected by UAP1 knockdown, which included various amino acids, organic acids, and fatty acids, suggesting that their concentrations are regulated by UAP1 metabolism (Figure 4G). Moreover, pathway enrichment analysis *via* Kyoto Encyclopedia of Genes and Genomes (KEGG) revealed that UAP1 knockdown significantly impacted several metabolic processes, including arginine synthesis and metabolism, phenylalanine synthesis and metabolism, and alanine-aspartate-glutamate metabolism (Figure 4H). These findings indicate that UAP1 plays a crucial role in the metabolic regulation of multiple amino acids *in vivo*. In summary, our study confirms the role of UAP1 knockdown in modulating tumorigenicity in MM cells and provides preliminary insights into its effects on the metabolic landscape of tumor tissues.

### 3.5 Transcriptomics and integrative analysis of UAP1 in MM

To further investigate the mechanisms by which UAP1 knockdown suppresses *in vivo* growth of MM, we performed transcriptomic analysis on the tumor tissues. Our initial findings indicated a trend of separation between the control and UAP1-KD groups, suggesting that UAP1 knockdown induces significant changes at the transcriptional level (Figure 5A). Specifically, volcano plots were generated to visualize differentially expressed genes, applying criteria of a |fold change| greater than five and an adjusted P-value less than 0.05 for gene selection. This stringent fold change threshold was chosen to prioritize core genes with stronger functional effects, thereby robustly reflecting the biological impact of UAP1 knockdown. This analysis revealed 523 upregulated genes and 100 downregulated genes in the UAP1-KD group, with the remaining genes showing no significant changes (Figure 5B). Further refinement led us to select the top 50 genes based on fold change rankings, which we then visualized using heat maps and clustering analysis (Supplementary Figure S7A). The changes in upregulated genes following UAP1 knockdown were particularly pronounced, with these genes implicated in various biological functions including protein digestion and absorption, protein binding, and hydrolase activity. Gene Ontology (GO) enrichment analysis indicated that the differentially expressed genes predominantly play roles in transcriptional modification and signal regulation, with localization primarily in the cell membrane and extracellular matrix, and are involved in molecular functions such as metal ion binding and transcription factor activity (Figure 5C). Additionally, KEGG pathway enrichment analysis identified significant alterations and gene enrichment in pathways including hematopoietic cell lineage, ECM-receptor interaction, protein digestion and absorption, and PI3K-Akt signaling pathway (Figure 5D). These findings were corroborated by Gene Set Enrichment Analysis (GSEA), which confirmed a significant decrease in genes associated with the protein secretion pathway in the UAP1-KD group. Notably, we observed that the G2M checkpoint pathway exhibited the significant impact in the UAP1-KD group, with relevant genes reduced in that cohort (Supplementary Figure S7B). Overall, these results demonstrate that UAP1 knockdown alters the gene expression profile, with the selected differentially expressed genes being associated with biological processes such as protein digestion and absorption, as well as protein binding. We hypothesize that UAP1-mediated O-GlcNAc modification may play a critical role in these processes.

**FIGURE 5:**
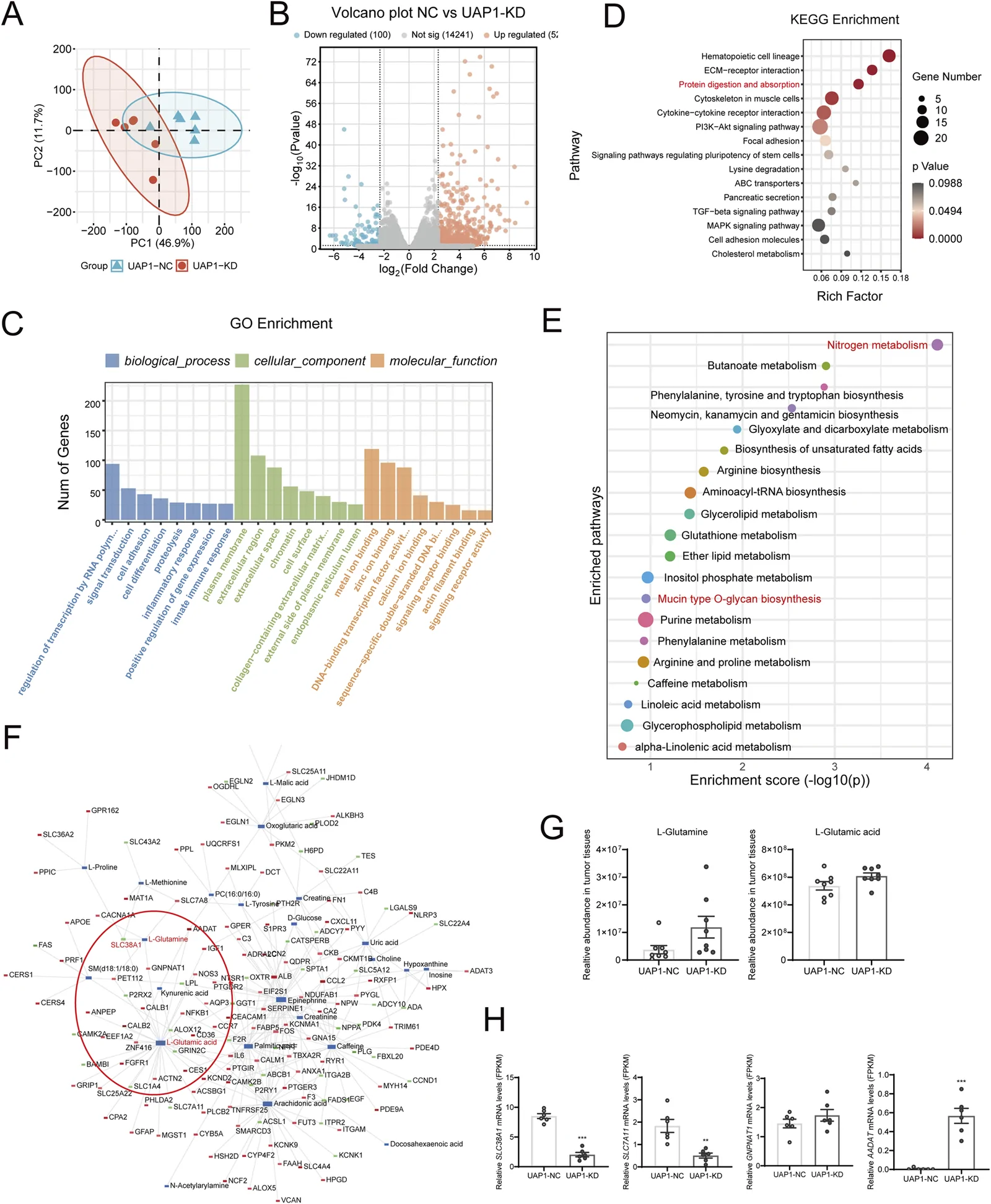
Transcriptomics and integrative analysis of UAP1 in MM. **(A)** PCA of transcriptomic data from mouse tumor tissues, comparing UAP1-NC and UAP1-KD groups. **(B)** Volcano plot illustrating the comparison of UAP1-NC and UAP1-KD groups from the transcriptomic analysis. **(C)** GO enrichment analysis of the differentially expressed genes. **(D)** KEGG pathway enrichment analysis of the differentially expressed genes. **(E)** Enrichment dot of pathway analysis based on integrative metabolomics and transcriptomics data. **(F)** Metabolite-gene correlation network analysis. **(G)** Semi-quantitative analysis of L-Glutamine and L-Glutamic acid in tumor metabolomics. **(H)** Expression alterations of SLC38A1 and SLC7A11 (genes related to L-Glutamine and L-Glutamic acid metabolism), GNPNAT1 (gene related to the HBP pathway), and AADAT (gene related to nitrogen metabolism) in tumor transcriptomics. Data are shown as mean ± SEM; *P < 0.05, **P < 0.01, ***P < 0.001, as assessed with an unpaired two-sided t-test.

To investigate the metabolites and genes affected by UAP1 knockdown, we performed an integrated analysis of metabolomics and transcriptomics data. Nitrogen metabolism was identified as the most significantly altered pathway, indicating that UAP1-mediated HBP perturbation is directly linked to nitrogen homeostasis. Additionally, mucin type O-glycan biosynthesis, a downstream pathway of the HBP, was significantly enriched, confirming that UAP1 knockdown disrupts both the HBP and its downstream signaling cascades (Figure 5E). Further in-depth analysis revealed strong associations between L-Glutamine and L-Glutamic acid and the transporter genes SLC38A1 and SLC7A11 (Figure 5F). Following UAP1 knockdown, intracellular levels of L-Glutamine and L-Glutamic acid were elevated (Figure 5G), whereas the expression of SLC38A1 and SLC7A11 was downregulated. Notably, GNPNAT1, a key enzyme in the HBP, showed increased expression, suggesting a compensatory feedback activation of the HBP, which also accounts for the observed elevation of UDP-hex in UAP1-knockdown MM cells. Moreover, AADAT, a gene involved in nitrogen metabolism, was significantly upregulated, indicating that the tumor nitrogen cycle enters a highly compensatory state upon UAP1 knockdown (Figure 5H). Given that L-Glutamine serves as the primary nitrogen source and carrier, participating in the urea cycle and amino acid synthesis while also acting as the core substrate of the HBP, it functions as a critical hub connecting the HBP and nitrogen metabolism. Therefore, UAP1 knockdown leads to reduced glutamine consumption, concomitant glutamic acid accumulation, nitrogen metabolic reprogramming, and suppressed expression of their transporters, resulting in decreased uptake. These findings collectively demonstrate that UAP1 is involved in nitrogen metabolism and validate the glutamine addiction of MM.

### 3.6 Molecular docking drug screening based on UAP1 and combination therapy

The identification of UAP1 as a therapeutic target has opened new avenues for the development of treatment strategies for MM. Building on our understanding of the biological functions and mechanisms of UAP1, we initiated a drug screening campaign aimed at identifying compounds that target this protein. Utilizing the ZINC and DrugBank databases, we compiled a list of FDA-approved small molecules and employed the LibDock module of Discovery Studio for virtual screening, followed by molecular docking using the CDOCKER module. We ranked the compounds based on their LibDock scores, identifying the top ten candidates, which included amsacrine, aminopterin, cefmetazole, moperone, and RAB (Table 1). Notably, our preliminary screening revealed that aminopterin and RAB exhibited potent effects on MM cells at nanomolar concentrations (Figure 6A). Given that aminopterin and amsacrine are already established in the treatment of lymphoma and myeloma respectively, they have limited potential for novel therapeutic insights in MM. In contrast, RAB, a proton pump inhibitor (PPI) with a well-documented safety profile, presents an innovative option for MM research. Subsequently, we investigated the efficacy of RAB on various MM cell lines, demonstrating that it significantly inhibited cell viability (Figure 6B). Molecular docking studies indicated that RAB forms hydrogen bonds with UAP1, suggesting a direct interaction between the drug and the target protein (Figure 6C). SPR analysis demonstrated a dose-dependent increase in response signals over time across the tested concentration gradients. The dissociation phase exhibited a normal declining trend, and no abnormal signals associated with baseline drift or non-specific binding were observed (Figure 6D). The KD value for the binding interaction between RAB and UAP1 was 96 μM, confirming a direct physical interaction. To evaluate the functional impact of the binding between RAB and UAP1, we first examined changes in UAP1 expression following RAB treatment at different time points and confirmed that UAP1 expression was markedly downregulated (Figures 6E,F). Furthermore, to investigate whether RAB exerts its effect through the enzymatic activity of UAP1, we measured intracellular UDP-GlcNAc levels in MM cells and found that RAB treatment at various time points did not alter the magnitude of UDP-GlcNAc elevation (Figure 6G), and this increase in UDP-GlcNAc was consistent with the previously observed elevation of UDP-hex upon UAP1 knockdown. Therefore, these results do not support the notion that RAB inhibits UAP1 enzymatic activity.

**TABLE 1:** Top 10 compounds sorted by LibDock score.

Num	Index	Generic name	Formula	Molecular weight	LibDock score
1	2,117	Sulfoxone	C_14_H_16_N_2_O_6_S_3_	404.482	153.447
2	14,291	Aminopterin	C_19_H_20_N_8_O_5_	440.413	147.331
3	383	Cefmetazole	C_15_H_17_N_7_O_5_S_3_	471.534	145.508
4	17,085	Moperone	C_22_H_26_FNO_2_	355.453	144.786
5	2,075	Rabeprazole	C_18_H_21_N_3_O_3_S	359.443	144.08
6	9,492	Oglufanide	C_16_H_19_N_3_O_5_	333.344	141.535
7	18,664	Ciforadenant	C_20_H_21_N_7_O_3_	407.434	140.395
8	8,540	Zidovudine	C_10_H_15_N_5_O_10_P_2_	427.201	140.135
9	15,766	Resminostat	C_16_H_19_N_3_O_4_S	349.41	140.066
10	18,095	Remetinostat	C_16_H_21_NO_6_	323.345	138.146

**FIGURE 6:**
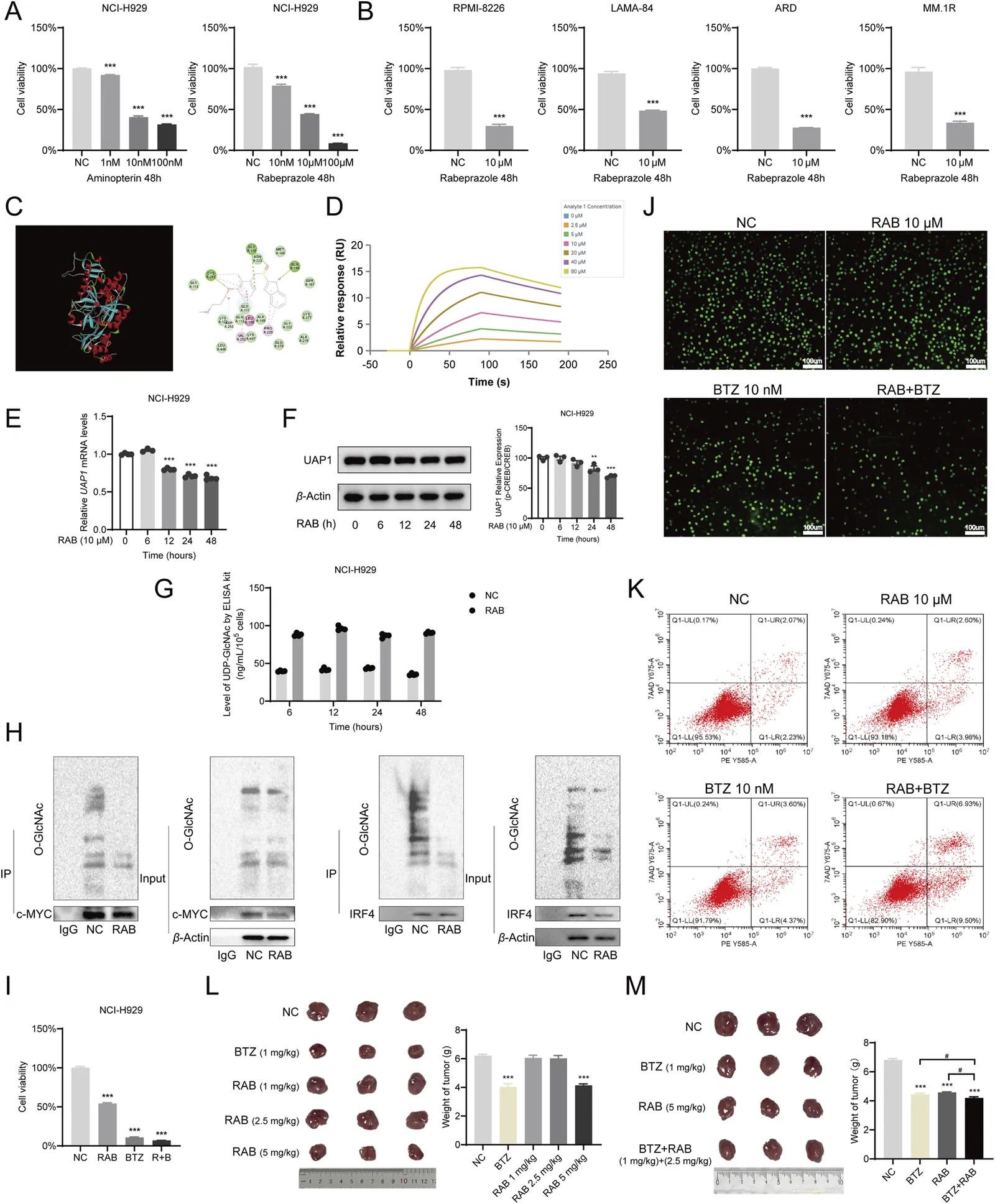
Anti-proliferative effects of RAB in MM and its enhanced activity with BTZ. **(A)** Cell viability in NCI-H929 cells following treatment with aminopterin and RAB after 48 h. **(B)** Evaluation of the inhibitory effects of RAB on MM cell lines after 48 h. **(C)** Molecular docking analysis of UAP1 and RAB. **(D)** SPR analysis of UAP1 and RAB. **(E)** Real-time qPCR analysis of *UAP1* mRNA expression in NCI-H929 cells following RAB treatment (10 μM) at various times. **(F)** Western blot analysis of UAP1 protein expression in NCI-H929 cells following RAB treatment (10 μM) at various times. **(G)** Intracellular UDP-GlcNAc levels measured by ELISA in NCI-H929 cells treated with RAB (10 μM) for different durations, normalized to cell count. **(H)** Co-IP of c-MYC and IRF4 by Western blot analysis of O-GlcNAc modification in NCI-H929 cells treated with 10 μM RAB for 48 h. **(I)** Cell viability in NCI-H929 cells to detect the cytotoxic effects of RAB (10 μM) combined with BTZ (10 nM) after 48 h. **(J)** EdU assay to detect the effect of RAB combined with BTZ on cell proliferation in NCI-H929 cells after 48 h. **(K)** PE/7-AAD assay to detect the effect of RAB combined with BTZ on cell apoptosis in NCI-H929 cells after 48 h. **(L)** Gross images of tumors in NOG mice subcutaneously implanted with NCI-H929 cells after administration of different doses of RAB. **(M)** Gross images of tumors *in vivo* after administration of RAB combined with BTZ. Data are shown as mean ± SEM; n ≥ 3 *P < 0.05, **P < 0.01, ***P < 0.001, as assessed with a one-way ANOVA test.

Since UDP-GlcNAc levels did not decrease as expected upon UAP1 suppression, this phenomenon may be attributed to compensatory activation of the HBP pathway. Accordingly, we hypothesized that the impact of UAP1 on UDP-GlcNAc might be less pronounced than its effect on O-GlcNAcylation. We collected c-MYC and IRF4 proteins, which are core targets driving MM proliferation, after RAB treatment and found that O-GlcNAc modification of both proteins was markedly suppressed (Figure 6H). These findings indicate that UAP1 expression inhibition not only suppresses global cellular O-GlcNAcylation levels but also directly influence O-GlcNAc-mediated protein modification. Additionally, our findings revealed that RAB can be effectively combined with BTZ, enhancing the inhibitory effects on cell viability, proliferation, and apoptosis (Figures 6I–K). When administered together, RAB and BTZ exhibited a more pronounced suppression of MM growth compared to each agent alone (Supplementary Figures S8A–C). The CI value of RAB combined with BTZ was 0.488, indicating synergistic effects between the two agents (Supplementary Figure S8D). *In vivo* validation of this combination further demonstrated that RAB exerted a dose-dependent inhibitory effect on the tumorigenicity of MM cells, with the combination treatment yielding significantly greater tumor suppression (Figures 6L,M). These results underscore the potential of targeting UAP1 with RAB as a promising therapeutic strategy for the treatment of MM, particularly in conjunction with BTZ.

### 3.7 UAP1 expression levels determine the sensitivity of MM cells to RAB

To further confirm the specificity of RAB in targeting UAP1 and to address the functional consequences of UAP1 modulation, we performed both knockdown and overexpression experiments in NCI-H929 cells. First, we investigated the effect of RAB intervention subsequent to UAP1 knockdown. EdU assay showed that both UAP1 knockdown and RAB treatment significantly suppressed cell proliferation, whereas the inhibitory effect of their combination was comparable to that of UAP1 knockdown alone (Figure 7A). Flow cytometric analysis indicated that while both siUAP1 and RAB notably increased the proportion of apoptotic cells, no attenuation of apoptosis was detected in the combined treatment group, and the apoptosis rate was similar to that observed under UAP1 knockdown alone (Figure 7B). Additionally, siUAP1 induced cell cycle arrest at the G1 phase, whereas RAB had a minimal impact on the cell cycle, primarily exerting its effects through the direct induction of MM cell apoptosis. However, the cell cycle was again arrested at the G1 phase (Figure 7C). More importantly, we conducted rescue experiments using a UAP1 overexpression (UAP1-OE) model to verify whether the anti-MM effects of RAB are specifically mediated through suppression of UAP1 expression. The expression of UAP1 was effectively validated upon treatment with UAP1 overexpression plasmid and RAB (Figure 7D). The EdU assay demonstrated that UAP1-OE enhanced the proliferative capacity of MM cells and, crucially, partially rescued the suppression of MM induced by RAB (Figure 7E). Similarly, while RAB treatment significantly elevated apoptosis levels, the overexpression of UAP1 significantly attenuated RAB-induced cell death (Figure 7F). However, the UAP1-OE group did not show significant changes in the distribution of the cell cycle, even in the case of RAB (Figure 7G). Taken together, these loss-of-function and gain-of-function experiments provide compelling evidence that UAP1 is a functional driver of MM cell growth and a specific therapeutic target of RAB. The ability of UAP1 overexpression to counteract the effects of RAB confirms that the drug’s anti-tumor activity is, at least in part, dependent on its modulation of the UAP1 pathway.

**FIGURE 7:**
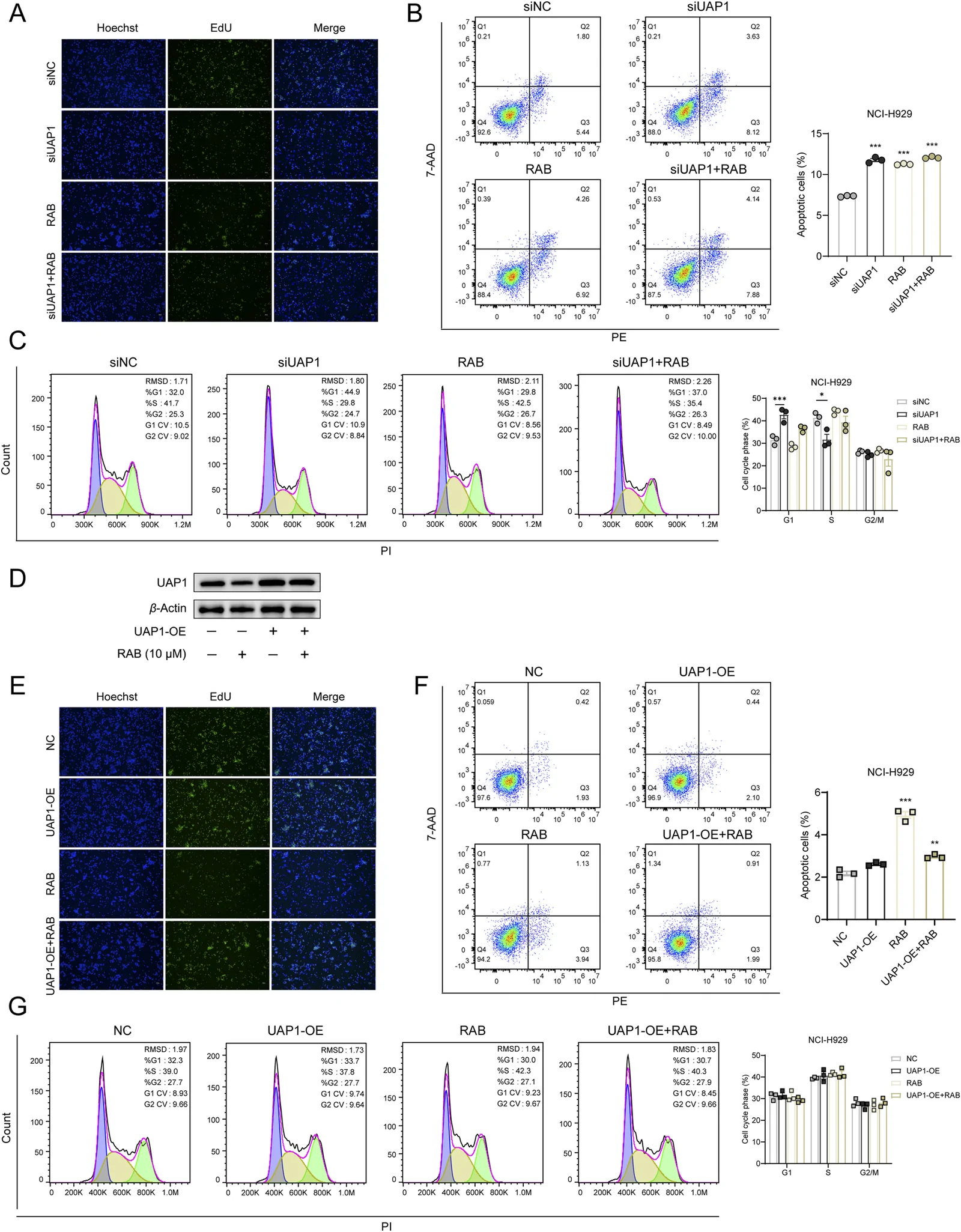
RAB inhibits the proliferation of MM cells by suppressing the UAP1 expression. **(A)** Cell proliferation of NCI-H929 cells after siUAP1+RAB (10 μM) treatment for 48 h detected by EdU assay. **(B)** Apoptosis of NCI-H929 cells after siUAP1+RAB (10 μM) treatment for 48 h detected by PE/7-AAD assay. **(C)** Cell cycle of NCI-H929 cells after siUAP1+RAB (10 μM) treatment for 48 h detected by PI assay. **(D)** Western blot analysis of UAP1 expression after UAP1-OE + RAB (10 μM) treatment in NCI-H929 cells. **(E)** Cell proliferation of NCI-H929 cells after UAP1-OE + RAB (10 μM) treatment for 72 h detected by EdU assay. **(F)** Apoptosis of NCI-H929 cells after UAP1-OE + RAB (10 μM) treatment for 72 h detected by PE/7-AAD assay. **(G)** Cell cycle of NCI-H929 cells after UAP1-OE + RAB (10 μM) treatment for 72 h detected by PI assay. Data are shown as mean ± SEM; n ≥ 3 *P < 0.05, **P < 0.01, ***P < 0.001, as assessed with a one-way ANOVA test.

To further investigate whether the anti-MM effects observed are a class-wide property of PPIs, we utilized omeprazole (OME), another representative PPI, as a parallel control. Our results demonstrated that OME had negligible inhibitory effects on the proliferation and apoptosis of MM cells at the concentrations where RAB was highly effective. Significant suppression of MM cell growth by OME was only observed at substantially higher concentrations (≥100 μM) (Supplementary Figure S9A–D). In contrast, OME inhibited UAP1 mRNA expression without suppressing UAP1 protein expression (Supplementary Figure S10A and B), which is fundamentally distinct from the effect of RAB. Since both RAB and OME belong to the PPI class, we sought to investigate whether their differential effects on UAP1 expression were mediated through the H^+^/K^+^-ATPase. We first examined the expression of ATP4A and ATP4B in MM cells; however, due to their extremely low transcript levels, neither gene could be reliably detected by real-time qPCR (data not shown). We subsequently assessed protein expression and found that ATP4A was undetectable, whereas ATP4B was present at minimal levels in normal B cells and expressed at even lower levels in MM cells (Supplementary Figure S10C). These comparative data suggest that the potent anti-MM activity of RAB is not merely a generic PPI-mediated effect but is significantly linked to its specific targeting of UAP1.

## 4 Discussion

In this study, we report that high expression levels of UAP1 are indicative of poor prognosis in MM patients. UAP1 enhances MM cell viability by regulating O-GlcNAcylation, and its knockdown effectively suppresses rapid cell proliferation. Mechanistically, UAP1 modulates amino acid metabolism, protein digestion and absorption, and GPI anchor biosynthesis, thereby influencing the metabolic and transcriptional fates of MM cells. To develop a therapeutic strategy targeting UAP1, we conducted drug screening and identified RAB as an expression suppressor of UAP1. Consequently, RAB has been proposed as a potential therapeutic agent for MM. This innovative application of RAB exemplifies a repurposing of “old” drugs and holds promising practical applications. Overall, this study explores the potential of UAP1 as a therapeutic target in MM, providing a comprehensive validation of its biological functions and elucidating the interaction between UAP1 and RAB, thus highlighting the potential use of RAB as a treatment for MM.

Targeted therapy is emerging as a novel approach in oncology, with drug targets serving as the central elements (Liu et al., 2021). Consequently, the development of these targets has become a focus in cancer research. Despite the continuous introduction of innovative therapies for MM, a clear understanding of the underlying therapeutic mechanisms remains elusive. Investigating potential drug targets for MM is essential for addressing relevant clinical challenges. This study explores the potential of UAP1 as a therapeutic target in MM. In fact, UAP1 has also been identified as a driver in prostate cancer, where its high expression correlates with androgen receptor levels and confers protection against endoplasmic reticulum stress (Itkonen et al., 2015). Furthermore, omics-based studies have confirmed that elevated UAP1 expression in bladder cancer and lung adenocarcinoma is associated with poor clinical outcomes, marking UAP1 as a promising therapeutic target (Wang et al., 2020; Puttamallesh et al., 2020). UAP1L1, a homolog of UAP1 with 59% identical amino acid sequences, demonstrates a high degree of similarity to UAP1. Wu et al. have shown that UAP1L1 is also a critical pathogenic factor in prostate cancer, contributing to disease progression (Wu et al., 2022). Similarly, UAP1L1 expression is significantly upregulated in hepatocellular carcinoma, mediating rapid cell proliferation and correlating with increased prognostic risk for patients (Lai et al., 2019). The findings concerning UAP1 collectively suggest its potential as a therapeutic target in cancer. Although the current literature on UAP1 is limited, our study establishes a direct correlation between elevated UAP1 expression and MM, thereby providing a robust theoretical foundation for further investigation into the biological roles of UAP1.

UAP1 plays a crucial role in the HBP, which is essential to cancer metabolism (Kim et al., 2020). As a key metabolic enzyme downstream in the HBP, UAP1 facilitates the synthesis of UDP-GlcNAc, a substrate necessary for O-GlcNAcylation. This pathway utilizes glutamine, the enzyme glutamine-fructose-6-phosphate amidotransferase, acetyl-CoA, and uridine triphosphate to generate UDP-GlcNAc (Le Minh et al., 2023). Under the catalytic action of O-GlcNAc transferase (OGT), the GlcNAc moiety of UDP-GlcNAc is linked to serine or threonine residues on target proteins, resulting in O-GlcNAcylation, which is known to modulate protein function, stability, and interactions (Soria et al., 2022; Forma et al., 2014). We hypothesize that UAP1’s regulation of the HBP also affects cellular O-GlcNAcylation processes. To test this, we measured UDP-GlcNAc levels and O-GlcNAcylation in MM cells and found that UAP1 expression directly influences the O-GlcNAcylation process. Specifically, interference with UAP1 led to a reduction in O-GlcNAcylation, a phenomenon previously reported in breast cancer, where the phosphorylation of UAP1 enhances its activity, positively impacting O-GlcNAcylation in breast cancer cells (Mason et al., 2020). O-GlcNAcylation is involved in critical cellular metabolic and signaling processes; for instance, after modification by UDP-GlcNAc, USP16 undergoes glcnacylation that inhibits its phosphorylation and maintains deubiquitination of H2A and PLK1, thereby regulating the cell cycle (Zhao et al., 2024).

In our findings, UAP1 interference significantly affected protein digestion, absorption, biosynthesis, and amino acid metabolism, indicating that UAP1 mediates cellular O-GlcNAcylation, a critical reversible post-translational modification belonging to the O-linked glycosylation family, features the attachment of a single N-acetylglucosamine moiety to protein serine/threonine residues, and modulates protein function and cellular signaling processes. When UAP1 expression is disrupted, alterations in the synthesis and metabolic processes of glycoproteins in MM cells are evident. Disturbances in O-GlcNAcylation have been associated with changes in amino acid metabolism and nutrient signaling; for example, OGT knockout has been shown to enhance the metabolism of multiple amino acids, such as serine and threonine, highlighting OGT’s role as a nutrient sensor (Li et al., 2019). The fluctuations in the metabolic substrate UDP-GlcNAc are particularly sensitive to changes in amino acid metabolism, which may explain how UAP1 knockdown and subsequent UDP-GlcNAc reduction affect amino acid metabolism in MM cells. From a therapeutic perspective, O-GlcNAcylation has emerged as a promising target for cancer treatments, as it integrates signaling pathways to influence genomic stability, epithelial-mesenchymal transition, apoptosis, and the cell cycle (Zhao et al., 2024; Hu et al., 2019; Efimova et al., 2019; Du et al., 2024; Pellegrini et al., 2023). Currently, strategies aimed at targeting O-GlcNAcylation are in the preclinical research phase, with several novel O-GlcNAcylation inhibitors demonstrating promising antitumor efficacy (Zhang et al., 2024; Lee et al., 2023). Therefore, continued exploration of O-GlcNAcylation in the context of MM is warranted.

Targeted drug development is a critical component of cancer treatment. Given the absence of specific UAP1 expression suppressor, our study initiated a virtual screening approach to identify potential compounds targeting UAP1, ultimately narrowing our focus to RAB. As a PPI commonly used for gastrointestinal disorders, the safety and cost-effectiveness of RAB are well-established (Baldwin and Keam, 2009); however, its efficacy in treating MM remains to be elucidated. Our findings provide experimental evidence supporting the use of RAB as a suppressor of MM, with preliminary studies confirming that its administration in mice does not result in any adverse effects on vital signs. Furthermore, we demonstrated that RAB directly targets UAP1 in MM cells and inhibits its expression. The mechanistic connection between RAB and UAP1 was also directly explored. It was observed that the effect of RAB was attenuated in UAP1-knockdown cells. Similarly, overexpression of UAP1 abolished the inhibitory effect of RAB, suggesting that this drug exerts its action primarily by disrupting UAP1-mediated processes. We also assessed UAP1 enzymatic activity by measuring UDP-GlcNAc levels and demonstrated that RAB binding to UAP1 does not alter its catalytic activity but solely suppresses its expression. Interestingly, when we combined RAB with BTZ, we observed a enhanced effect, which is particularly relevant given the need to enhance the therapeutic efficacy of proteasome inhibitors in MM patients. The clinical significance of novel targets like UAP1 can be evaluated on this background, because the emergence of relapsed/refractory MM remains a critical clinical challenge. Our validation in an independent sample cohort confirms the significant upregulation of UAP1 in MM. Notably, the enhanced effect observed between RAB and BTZ indicates that UAP1-targeted strategy could potentially sensitize MM cells to proteasome inhibitors. The synergistic anti-myeloma effect observed between RAB and BTZ can be attributed to the convergence of metabolic stress and proteotoxic stress. BTZ inhibits the 26S proteasome, leading to the accumulation of misfolded proteins and activation of the unfolded protein response. Suppression of UAP1 expression concurrently reduces O-GlcNAcylation, impairing protein quality and lowering the threshold for ER stress-induced apoptosis. Furthermore, suppression of UAP1 expression triggers glutamine metabolic reprogramming, damaging the proteasomal degradation. The combined action indicates a “double-hit” where both the capacity to fold proteins and the ability to degrade them are damaged.

The rationale for targeting UAP1 in MM is the selective dependency on hyperactivated HBP. Normal hematopoietic cells maintain basal HBP activity to support physiological O-GlcNAcylation, whereas MM cells showed UAP1 upregulation, creating a targetable metabolic vulnerability. Notably, UAP1 homozygous knockout is embryonically lethal in mice, and heterozygous mice exhibit impaired antiviral immunity, underscoring the physiological importance of UAP1 (Yang et al., 2023). However, our data show markedly lower UAP1 expression in non-malignant bone marrow cells relative to MM cells, indicating preferential sensitivity of malignant cells to UAP1 suppression. Regarding potential toxicities, repurposing RAB offers safety advantages given its well-documented clinical safety. Although the concentration of 10 μM used in our *in vitro* study exceeds the reported human plasma Cmax of approximately 1.13 μM under standard oral dosing, our *in vivo* data confirms that RAB exerts significant tumor suppression at tolerable doses. The enhanced activity observed between RAB and BTZ suggests that UAP1 suppression can enhance the efficacy of monotherapy, potentially reducing the required dose for each agent. The moderate UAP1 expression suppression by RAB at clinically achievable exposures, combined with the selective HBP addiction of MM cells, supports the existence of a viable therapeutic window that warrants further preclinical and clinical investigation. Notably, long-term RAB use is associated with potential risks, including elevated incidence of fractures and infection. These concerns are particularly relevant for the predominantly elderly MM population. For clinical translation, we propose risk-mitigation strategies: intermittent dosing schedules, routine bone density monitoring, and prophylactic calcium plus vitamin D supplementation, to balance antitumor efficacy with long-term tolerability.

There are other areas in this research that warrant improvement. It is important to acknowledge the limitations of the *in vivo* model used in this study. We primarily relied on a subcutaneous xenograft model to evaluate the anti-MM effects of UAP1 suppression and RAB treatment. This model offers several practical advantages, including minimal individual variability, direct tumor visualization and quantification, and rapid modeling, all of which facilitate efficient preclinical drug screening. While this model provided clear evidence of tumor growth suppression, it fails to fully recapitulate the complex and specialized BM microenvironment. Therefore, the efficacy of targeting UAP1 needs to be further validated. Additionally, the clinical sample size used for UAP1 expression validation was relatively small, which may limit the generalizability of our clinical findings. Future studies with expanded patient cohorts are planned to further validate UAP1 expression levels and their clinical significance in MM. Orthotopic xenograft models were not selected for the present study, however, they are under consideration for our future investigations. Such models enable us to evaluate the UAP1 targeted therapies within the authentic bone marrow microenvironment, serving as an alternative strategy for verification. Furthermore, it is important to acknowledge that PPIs have been reported to exert non-specific anti-tumor effects across various cancers through mechanisms such as lysosomal alkalinization, inhibition of V-ATPase, and modulation of autophagy (Marino et al., 2010; Bridoux et al., 2022). However, our study distinguishes the UAP1-specific therapeutic strategy from these class-wide PPI effects. The comparative analysis revealed that OME exhibited significantly lower potency against MM cells compared to RAB at identical concentrations. This disparity underscores that the mechanism of RAB is the disruption of the UAP1-mediated HBP. Specifically, the fact that OME, another representative PPI, failed to suppress UAP1 protein expression and showed negligible anti-MM effects at equivalent concentrations further supports that RAB’s anti-MM activity is primarily mediated through UAP1 rather than through proton pump inhibition or other PPI class-wide effects. While the binding affinity of RAB for UAP1 did not reach the low-micromolar range, its observed inhibitory activity has prompted our ongoing efforts toward the structural optimization of RAB. Engineering next-generation UAP1 expression suppressors with enhanced binding potency represents a compelling avenue for future investigation. Finally, mechanistic limitations regarding the RAB-UAP1 interaction warrant further clarification. Specially, while we identified global changes in protein O-GlcNAcylation, the specific downstream oncogenic substrates whose glcnacylation status is most critical for MM cell survival under UAP1 regulation remain unknown. Future glycoproteomic investigations are needed to map these specific effectors and fully elucidate the therapeutic landscape of UAP1 expression suppression.

## 5 Conclusion

Our study uncovers UAP1’s function in MM and its association with HBP. Additionally, we demonstrated for the first time that RAB, a PPI, exhibits anti-tumor effects in MM. While the clinical application of these findings requires further optimization regarding dosing and delivery, our work offers novel insights and preclinical foundation for RAB repurposing and MM treatment.

## Data Availability

The original contributions presented in the study are publicly available. This data can be found here: https://doi.org/10.5061/dryad.g79cnp66t.

## Supplementary material

SUPPLEMENTARY FIGURE 1 The relationship between ATP8B2 expression and MM patients from database analysis. **(A)** MMRF database showed the relationship between ATP8B2 expression and patient survival, P<0.001. **(B–D)** The GEO database was used to analyze ATP8B2 expression across different stages and phases of MM.

SUPPLEMENTARY FIGURE 2 The relationship between AQP3 expression and MM patients from database analysis. **(A)** MMRF database showed the relationship between AQP3 expression and patient survival, P<0.05. **(B–D)** GEO database analyzed the expression of AQP3 in different stages and phases of MM.

SUPPLEMENTARY FIGURE 3 The relationship between CADPS2 expression and MM patients from database analysis. **(A)** MMRF database showed the relationship between CADPS2 expression and patient survival, P<0.05. **(B–D)** GEO database analyzed the expression of CADPS2 in different stages and phases of MM.

SUPPLEMENTARY FIGURE 4 The relationship between JCHAIN expression and MM patients from database analysis. **(A)** MMRF database showed the relationship between JCHAIN expression and patient survival, P<0.05. **(B,C)** GEO database analyzed the expression of JCHAIN in different stages and phases of MM.

SUPPLEMENTARY FIGURE 5 The relationship between BMP6 expression and MM patients from database analysis. **(A)** MMRF database showed the relationship between BMP6 expression and patient survival, P=0.061. **(B–D)** GEO database analyzed the expression of BMP6 in different stages and phases of MM.

SUPPLEMENTARY FIGURE 6 The relationship between BMP8B expression and MM patients from database analysis. **(A)** MMRF database showed the relationship between BMP8B expression and patient survival, P<0.05. **(B–D)** GEO database analyzed the expression of BMP8B in different stages and phases of MM.

SUPPLEMENTARY FIGURE 7 Transcriptional mechanism of UAP1-KD suppressing MM tumor growth in vivo. **(A)** Heatmap displaying the top 50 differentially expressed genes ranked by fold change. **(B)** GSEA of the differentially expressed genes.

SUPPLEMENTARY FIGURE 8 Combined inhibitory effect of RAB and BTZ on NCI-H929 cells. **(A)** Cell viability after RAB treatment for 48 hours detected by CCK-8 assay. **(B)** Cell viability after BTZ treatment for 48 hours detected by CCK-8 assay. **(C)** Cell viability after RAB+BTZ treatment for 48 hours detected by CCK-8 assay. **(D)** CI values for the RAB and BTZ combination. Data are shown as mean ± SEM; n ≥ 3 *P < 0.05, **P < 0.01, ***P < 0.001, as assessed with a one-way ANOVA test.

SUPPLEMENTARY FIGURE 9 The pharmacological effect of OME on the proliferation of MM cells. **(A)** Cell viability of MM cells after OME treatment for 48 hours detected by CCK-8 assay. **(B)** Cell proliferation of MM cells after OME treatment for 48 hours detected by EdU assay. **(C)** Cell cycle of MM cells after OME treatment for 48 hours detected by PI assay. (D) Apoptosis of MM cells after OME treatment for 48 hours detected by PE/7-AAD assay. Data are shown as mean ± SEM; n ≥ 3 *P < 0.05, **P < 0.01, ***P < 0.001, as assessed with a one-way ANOVA test.

SUPPLEMENTARY FIGURE 10 Inhibition of UAP1 is a unique effect of RAB independent of the general pharmacological mechanism of PPIs. **(A)** Real-time qPCR analysis of UAP1 expression in MM cells treated with 10 μM OME for 48 hours. **(B)** Western blot analysis of UAP1 expression in MM cells treated with 10 μM OME for 48 hours. **(C)** Western blot analysis of two H^+^/K^+^-ATPase subunits (ATP4A and ATP4B) expression in normal B cells and MM cells.
